# ACE2-Decoy-Conjugated PLGA-PEG Nanoparticles Loaded with Nafamostat for Potent Antiviral Activity

**DOI:** 10.3390/v17091167

**Published:** 2025-08-27

**Authors:** Shulin Hou, Yunyun Zhang, Xin Zheng, Ruining Li, Taoran Zhao, Hua Qiao, Xiaozheng Zhang, Zhizhen Liu

**Affiliations:** 1 Shanxi Key Laboratory of Birth Defect and Cell Regeneration, MOE Key Laboratory of Coal Environmental Pathogenicity and Prevention, Department of Biochemistry and Molecular Biology, College of Basic Medicine, Shanxi Medical University, Taiyuan 030001, China; hou_shulin1@163.com (S.H.); yyzhang2023@126.com (Y.Z.); 15905665865@163.com (X.Z.); ruining_li2025@163.com (R.L.); zhaotaoran@sxmu.edu.cn (T.Z.); qiaohua0410@126.com (H.Q.); zhangxiaozheng@sxmu.edu.cn (X.Z.); 2 Academy of Medical Sciences, Shanxi Medical University, Taiyuan 030001, China

**Keywords:** SARS-CoV-2, ACE2 decoy, nafamostat, PLGA-PEG, nanoparticles

## Abstract

Angiotensin-converting enzyme 2 (ACE2) is a key mediator of SARS-CoV-2 host cell entry, making it an attractive target for drug delivery strategies. Nafamostat (NM), a multifunctional agent with antiviral and anti-inflammatory properties, holds promise for COVID-19 treatment. In this study, we developed PLGA-PEG nanoparticles encapsulating NM (NM-PP NPs) and further conjugated them with specific ACE2 decoys (CTC-445.2d or SI5α) to generate NM-PP-Pro/Pep NPs. Both unmodified and ACE2-decoy-modified NPs exhibited uniform size distributions (diameter < 200 nm) and negative surface charges, as confirmed by dynamic light scattering and zeta potential measurements. The nanoparticles maintained structural integrity for at least 18 days at 4 °C and room temperature. In vitro release studies revealed sustained and controlled NM release kinetics. Notably, NM-PP-Pro NPs displayed potent antiviral activity, with an IC_50_ < 0.05 nM against wild-type SARS-CoV-2 and remained effective against the D614G variant (IC_50_ = 2 nM). These results underscore the potential of NM-PP-Pro NPs as a versatile nanotherapeutic platform for targeting SARS-CoV-2 and its emerging variants.

## 1. Introduction

The World Health Organization (WHO) declared the end of the COVID-19 global health emergency in 2023; yet, SARS-CoV-2 continues to circulate, mutate, and evolve, remaining a significant threat to global public health [1,2,3]. Since the onset of the epidemic, extensive research efforts have focused on developing effective prevention and treatment strategies. While numerous vaccines have demonstrated efficacy in preventing infection and reducing severe outcomes [4,5,6,7,8], therapeutic interventions face substantial challenges. Currently approved antiviral drugs, including RNA polymerase inhibitor (e.g., Lagevrio) and main protease (Mpro) inhibitors (e.g., Paxlovid and Xocova) [9,10,11,12], are limited by the following factors: rapid viral mutations leading to frequent drug resistance and repeated viral rebound, and suboptimal pharmacokinetic properties resulting in inadequate target-site concentrations and adverse effects [13,14,15]. These limitations highlight the urgent need for innovative drug delivery systems and novel therapeutic approaches to combat COVID-19 effectively.

SARS-CoV-2 utilizes angiotensin-converting enzyme 2 (ACE2) as its primary host cell entry receptor, with viral attachment medicated by spike (S) protein binding [16,17,18]. Among the known human-infecting coronaviruses, HCoV-NL63, SARS-CoV, and SARS-CoV-2 predominantly rely on ACE2 receptors for cellular entry [19,20]. Viral fusion is further promoted by transmembrane serine protease 2 (TMPRSS2), which cleaves the S protein at the S2’ field to facilitate plasma membrane fusion. Alternatively, in TMPRSS2-deficient environments, the virus enters via endocytosis in a cathepsin-dependent manner [21]. This dual-entry mechanism underscores the central role of ACE2 and highlights TMPRSS2 as a facilitator of efficient fusion.

Given the critical role of ACE2 in viral pathogenesis, disrupting the S-ACE2 interaction presents a promising therapeutic strategy [22,23]. The design of “ACE2-mimetic” decoys provides a distinct advantage by neutralizing the virus while preserving endogenous ACE2 function [24]. This decoy strategy employs biomimetic designs that imitate natural host receptors to achieve the efficient and specific neutralization of pathogenic toxins. This approach demonstrates broad cross-disciplinary utility, as exemplified by targeted mCuS@lm nanocages in nanomedicine that neutralize bacterial toxins, mitigate cytokine storms, and selectively induce cuproptosis in drug-resistant bacteria without harming healthy cells [25]. Similarly, in agriculture, carboxylic acid-derived decoy substrates inhibit laccase virulence by active-site occupation, offering a sustainable path for eco-friendly pesticide development [26]. Efforts in “ACE2-mimetic” decoys have evolved from the early use of human recombinant soluble ACE2 [27,28] to more refined strategies such as engineered fragments [29,30], peptide mimetics [31,32,33], and optimized decoy receptors [34,35]. Notable examples include the rationally designed ACE2 decoys CTC-445.2d (IC_50_ < 5 nM) and peptide mimetics SI5α (IC_50_ = 1.59 μM), both of which show potent antiviral activity across multiple cell models [33,35]. ACE2-containing extracellular vesicles (EVs) inhibit SARS-CoV-2 infection more effectively than soluble ACE2 [36]. Another study developed ACE2 membrane-coated PLGA nanoparticles loaded with quercetin, demonstrating superior antiviral effects over free quercetin or uncoated nanoparticles [37]. Therefore, ACE2-based derivatives not only enhance antiviral potency but also improve biocompatibility and prolong circulation half-life, advancing their utility as drug delivery platforms.

Nafamostat (NM), a synthetic broad-spectrum serine protease inhibitor, exhibits potential as an anti-coronavirus therapeutic [38]. It effectively inhibits SARS-CoV-2 membrane fusion by targeting TMPRSS2, demonstrating 10-fold greater potency than the analogous drug Camostat [39,40,41]. Beyond its antiviral activity, NM exerts anticoagulant, anti-fibrinolytic, and anti-inflammatory effects [42,43,44], suggesting a multi-mechanistic role in COVID-19 treatment. However, its clinical utility is constrained by rapid in vivo hydrolysis, poor bioavailability, and venous inflammation following intravenous injection [45,46,47]. These challenges underscore the need for advanced drug delivery systems to improve both the efficacy and safety of NM.

In the present study, we first encapsulated NMinto PLGA-PEG nanoparticles (NM-PP NPs) and then functionalized the nanoparticles with two distinct ACE2 decoys. One of these is CTC-445.2d, a de novo designed decoy protein with nanomolar affinity for all three receptor binding domains of the S protein. Due to its ultrahigh stability and compact structure, CTC-445.2d outperforms native ACE2 in neutralizing SARS-CoV [35]. This decoy was conjugated to the NM-loaded nanoparticles to form CTC-NM-PP-Pro NPs. Additionally, we functionalized the NM-PP NPs with SI5α, a heptapeptide mimetic developed via a computer-aided approach based on the S protein binding domain of ACE2 [33], resulting in NM-PP-Pep NPs. We confirmed their favorable physicochemical parameters and anti-SAR-CoV-2 activity of these formulations in vitro, compared to the unmodified nanoparticles. This dual-functional strategy combines targeted drug delivery with receptor blockade, offering a novel approach to modulate virus–host interactions and providing a theoretical framework for developing antiviral therapeutic systems.

## 2. Materials and Methods

### 2.1. Protein Expression and Purification of Recombinant CTC-445.2d

The CTC-445.2d gene [35] was chemically synthesized (Shenzhen Kangti Life Technology Co., Ltd., Shenzhen, China) and inserted into the pET-29b (+) expression vector to construct the recombinant plasmid pET-29b (+)-CTC-445.2d. Subsequently, the recombinant plasmid was transformed into *Escherichia coli* BL21(DE3), and positive clones were selected on kanamycin-containing LB agar plates. For protein expression, a single positive colony was picked and inoculated into LB medium and cultured at 37 °C with shaking until the optical density at 600 nm (OD600) reached 0.6–0.8. Protein expression was then induced by the addition of 0.5 mM isopropyl-β-D-thiogalactopyranoside (IPTG, Shanghai yuanye Bio-Technology Co., Ltd., Shanghai, China), followed by low-temperature induction at 16 °C for 16 h. The bacterial cells were harvested by centrifugation and resuspended in lysis buffer (20 mM Tris-HCl, 500 mM NaCl, pH 8.0). Cell disruption was performed using a high-pressure cell disruptor (JN-Mini Pro crusher, Guangzhou Juneng Nano&Bio Technology Co., Ltd., Guangzhou, China. 1.2 MPa, 4 °C, three times), and the lysate was clarified by centrifugation at 12,000× *g* for 30 min at 4 °C. The supernatant was subsequently subjected to nickel-affinity chromatography purification. The chromatography column was pre-equilibrated with binding buffer (20 mM Tris-HCl, 500 mM NaCl, 20 mM imidazole, pH 8.0), and the target protein was eluted using an imidazole gradient. The eluted fractions were analyzed by 12% SDS-PAGE to verify the purity and molecular weight of aim protein. The aim protein with >90% purity was subjected to dialysis (PBS buffer) and subsequently stored at −80 °C for future use.

### 2.2. Production of Nafamostat-Loaded Nanoparticles

Nafamostat-loaded PLGA-PEG-COOH nanoparticles (NM-PP NPs) were prepared using a water-in-oil-in-water (*w*/*o*/*w*) double emulsion solvent evaporation method. In brief, 30 μL of nafamostat solution (MedChemExpress, Zhenghe Bio-Technology Co., Ltd., Taiyuan, China) was emulsified in 1 mL of dichloromethane containing 10 mg/mL PLGA15k-PEG3.4k-COOH (Xi’an Ruixi Biological Technology Co. Ltd., Xi’an, China) by ultrasonic homogenization (80% amplitude, 1 min) to form the primary emulsion (*w*/*o*). This emulsion was then dispersed in 6 mL of 1% (*w*/*v*) polyvinyl alcohol (PVA) aqueous solution and homogenized similarly to form the secondary emulsion (*w*/*o*/*w*). The resulting emulsion was transferred to 4 mL of 1% PVA solution and stirred (300 rpm) for 4 h to allow organic solvent evaporation. The nanoparticles were collected by centrifugation (13,000× *g*, 40 min, 4 °C, Eppendorf, Hamburg, Germany), washed with water, and then resuspended in 4 mL of water for storage at 4 °C.

### 2.3. Conjugation of ACE2 Decoy to NM-PP NPs

ACE2 decoy-modified nanoparticles (NM-PP-Pro NPs and NM-PP-Pep NPs) were prepared via 1-(3-Dimethylaminopropyl)-3-ethylcarbodiimide hydrochlorid (EDC)/N-Hydroxysuccinimide (NHS) chemistry as previously described [41,48]. Briefly, the prepared NM-PP NPs (5 mg) were resuspended in 1 mL of water, followed by the addition of 250 μL of EDC (1 mg/mL) and 250 μL of NHS (1 mg/mL), and then they were activated by stirring at 250 rpm at room temperature for 4 h. A 2 h coupling reaction was then performed with the addition of CTC-445.2d protein or SI5α peptide (sequence: QAKTFLD, synthesized by Nanjing Genscript Biotechnology Co., Ltd., Nanjing, China) in the concentration of 1 mg/mL. Uncoupled molecules and catalysts were removed by centrifugation (12,000× *g*, 20 min), resulting in ACE2 decoy-modified NM-PP NPs.

### 2.4. Nanoparticle Characterization

#### 2.4.1. Physicochemical Properties

The hydrodynamic diameter, polydispersity index (PDI), and zeta potential of nanoparticles including NM-PP NPs, NM-PP-Pro NPs, and NM-PP-Pep NPs were determined by dynamic light scattering (Malvern ZetaSizer Nano-ZSE, Malvern Panalytical, UK). The morphology of these nanoparticle formulations was observed using transmission electron microscopy (JEM-1230, Japan Electron Optics Laboratory Co., Ltd., Tokyo, Japan). Stability was assessed by monitoring particle size and PDI over 18 days at 4 °C and room temperature. A Fourier transform infrared (FT-IR) spectrometer (4000–500 cm^−1^) was acquired using a Bruker Invenio spectrometer (Bruker, Billerica, MA, USA) (4 cm^−1^ resolution, 32 scans).

#### 2.4.2. Encapsulation and Conjugation Efficiency

Nafamostat encapsulation efficiency (EE%) and drug loading (DL%) were calculated using Formulas (1) and (2), respectively. The amount of nafamostat was determined using ultraviolet spectrophotometry (Hitachi UH5300, Hitachi, Tokyo, Japan) at a wavelength of 242 nm:

(1) $$\left(EE\%\right)=\frac{Amount\;of\;encapsulated\;nafamostat}{Total\;input\;amount\;of\;nafamostat}$$

(2) $$\left(DL\%\right)=\frac{Amount\;of\;encapsulated\;nafamostat}{Weight\;of\;nanoparticles}$$

Conjugation efficiency (CE%) was calculated according to Formula (3) as follows, and protein concentration was determined by BCA assay (Omni-Easy™ BCA Kit, Epizyme, Shanghai, China):

(3) $$\left(CE\%\right)=\frac{Weight\;of\;protein\;in\;nanoparticles}{Total\;input\;amount\;of\;protein}$$

#### 2.4.3. In Vitro Drug Release

The in vitro nafamostat release study of NM-PP NPs was evaluated in the dialysis system, and an acetic-buffered solution was used as the release media [49,50]. NM-PP NPs and B-PP NPs were placed in a dialysis bag (MWCO 3 kDa), respectively, and then incubated in an acetic-buffered solution (10 mM NaAc, pH 5) at 37 °C, and stirred at 120 rpm. At specific time intervals, the release medium was replaced with the same volume of fresh medium. Samples (700 μL) were taken at predetermined time points (2, 4, 8, 12, 24, 36, 48, 60, 72, 84, 96, and 108 h). As described above, the concentration of nafamostat in the release medium was determined using UV spectrophotometry.

### 2.5. Biological Evaluation

#### 2.5.1. SARS-CoV-2 Pseudovirus Neutralization

SARS-CoV-2 pseudovirus neutralization assays were conducted using replication-incompetent lentiviral particles (Vazyme, Nanjing, China) bearing the SARS-CoV-2 spike glycoprotein and a firefly luciferase reporter gene. The following two pseudovirus variants were employed: the wild-type (DD1502–03) and the D614G mutant (DD1714–02). Assays were performed in human embryonic kidney 293T cells stably expressing ACE2 (Vazyme, DD1701–01) [51]. Prior to infection, pseudoviruses were diluted in DMEM to a working concentration of 1–2 × 10^4^ TCID_50_/mL. Serially diluted test samples (90 μL) were mixed with an equal volume of pseudovirus suspension and incubated at 37 °C for 1 h. Control wells included virus control (90 μL DMEM + 90 μL pseudovirus) and blank control (180 μL DMEM). Following incubation, 50 μL of each mixture or control was transferred to a 96-well white plate, followed by the addition of 50 μL of 293T-ACE2 cell suspension (2 × 10^4^ cells per well). At 20–24 h post-inoculation, 25 μL of DMEM supplemented with 10% FBS was added, and cells were cultured for an additional 24 h. Luciferase activity was then measured using Bio-Lite Luciferase Assay Reagent (Vazyme, DD1201–01). Relative luminescence units (RLU) were recorded on a SpectraMax^®^ iD5 microplate reader (Molecular Devices, Silicon Valley, CA, USA). The inhibition rate was calculated as follows: Inhibition (%) = [1 − (RLU_sample_ − RLU_blank_)/(RLU_virus_ − RLU_blank_)] × 100%. Dose–response curves were fitted and IC_50_ values derived using GraphPad Prism 10.

#### 2.5.2. Cytotoxicity Assay

Cell cytotoxicity was measured using the CCK-8 kit (Biosharp, Beyotime Biotechnology, Jingsu, China) according to the manufacturer’s protocol. Briefly, cells were seeded in 96-well plates at a density of 5 × 10^3^ cells/well and allowed to adhere for 24 h. Serial dilutions of test compounds were added to triplicate wells and incubated for an additional 24 h. Subsequently, 10 μL of CCK-8 solution was added to each well followed by incubation at 37 °C for 3 h. Absorbance was measured at 450 nm using a microplate reader (Molecular Devices). Cell viability (%) was calculated as follows: (ODtest − ODempty)/(ODcontrol − ODempty) × 100%.

#### 2.5.3. Cellular Uptake of Nanoparticles

During the preparation of nanoparticles, coumarin-6 (Solarbio, Beijing, Chnia) was added to the organic phase to prepare fluorescently labeled nanoparticles. Cellular uptake of coumarin-6 (C6)-labeled nanoparticles was evaluated in 293T cells and RAW264.7 seeded in 24-well plates (5 × 10^4^ cells/well). After 24 h of cell adhesion, the culture medium was replaced with C6-nanoparticles for 3 h at 37 °C. Cells were then washed three times with PBS, fixed with 4% paraformaldehyde (15 min) and nuclei-stained with DAPI (1 μg/mL, 10 min). Fluorescent images were captured using an inverted fluorescence microscope (Nikon Eclipse Ti, Nikon, Tokyo, Japan).

### 2.6. Statistical Analysis

All statistical analyses were conducted using the GraphPad Prism 10 (GraphPad Software Inc., San Diego, CA, USA). Data are presented as mean ± standard deviation (SD). For comparisons among multiple groups, one-way analysis of variance (ANOVA) followed by Tukey’s multiple comparisons test was applied. Statistical significance was defined as follows: * *p*< 0.05, ** *p*< 0.01, *** *p*< 0.001 and **** *p*< 0.0001.

## 3. Results

### 3.1. Preparation and Characterization of Nafamostat-Loaded Nanoparticles

**Figure 1:**
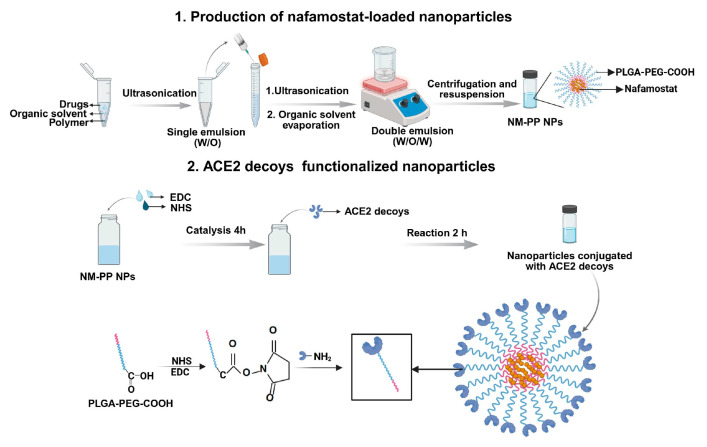
Schematic diagram of nafamostat-loaded ACE2 decoy-modified nanoparticles.

ACE2 decoy-modified nafamostat (NM)-loaded nanoparticles were prepared via a two-step process. First, NM-encapsulated poly lactic-co-glycolic acid-polyethylene glycol-carboxyl (PLGA-PEG-COOH) nanoparticles (NM-PP NPs) were synthesized using a water-in-oil-in-water (*w*/*o*/*w*) double-emulsion method. Subsequently, the NPs were functionalized with ACE2 decoys or peptides via carbodiimide-mediated conjugation (Figure 1).

**Figure 2:**
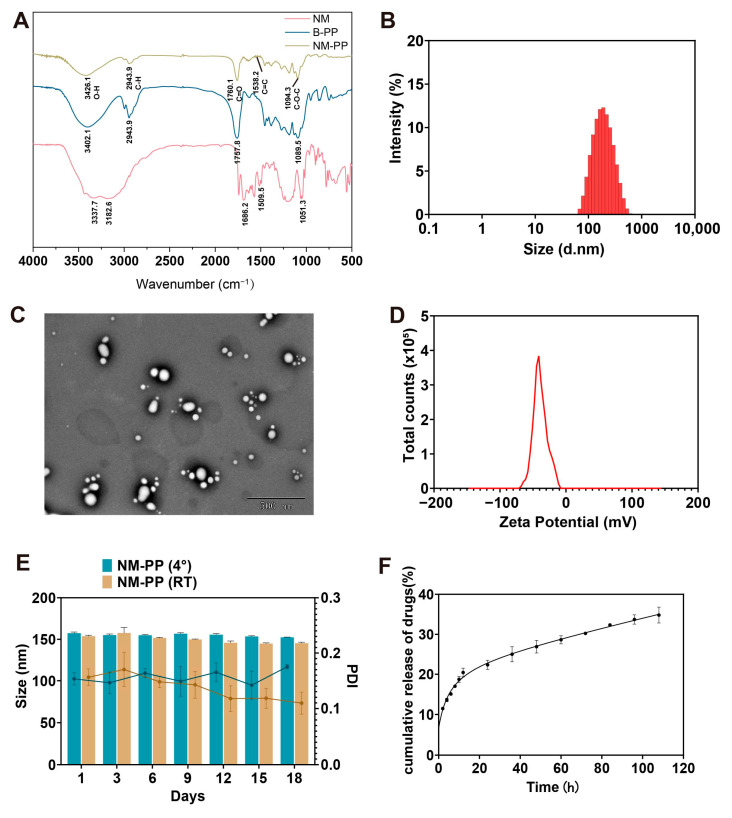
Physical characterization of nafamostat-loaded nanoparticles (NM-PP NPs). (**A**) FT-IR spectra of NM, B-PP NPs, and NM-PP NPs. (**B**–**D**) Hydrodynamic diameter distribution (**B**), TEM (**C**), and zeta potential (**D**) of NM-PP NPs. (**E**) The particle size changes of NM-PP NPs investigated after placed at 4 °C and RT for 18 days (*n* = 3). (**F**) In vitro release of NM-PP NPs (mean ± s.d., *n* = 3).

Following the preparation of NM-PP NPs using *w*/*o*/*w* double emulsion method, a Fourier transform infrared (FT-IR) spectrometer was employed to confirm the successful loading of NM into PP NPs, compared with bare PLGA-PEG-COOH NPs (B-PP NPs) and pure NM (Figure 2A). While most peaks of NM were obscured in the NM-PP NPs spectrum due to the dominant polymeric matrix signals, key spectral features confirmed the successful NM loading. Specifically, the presence of the NM aromatic ring skeleton vibration peak (~1538 cm^−1^), along with the copolymer hydroxyl (O-H) group stretching peak (~3426 cm^−1^) and the ester carbonyl (C=O) stretching peak (~1760 cm^−1^), verified successful NM incorporation into the PP NPs. Quantitative analysis by ultraviolet-visible (UV-Vis) spectrophotometry revealed favorable encapsulation parameters, with an encapsulation efficiency (EE) of 74.93 ± 2.18% and a drug loading capacity (DL) of 3.44 ± 0.17% in NM-PP NPs (Table 1).

Subsequent physicochemical characterization of the NM-PP NPs included the evaluation of particle size distribution, polydispersity index (PDI), surface potential, morphology, stability, and drug release kinetics (Table 1). Dynamic light scattering (DLS) analysis revealed that both B-PP NPs and NM-PP NPs exhibited narrow size distributions, with average hydrodynamic diameters of 155.4 ± 0.81 nm and 157.3 ± 1.43 nm (Table 1, Figure 2B), respectively. The low PDI value (<0.3) confirmed the monodisperse nature of the NM-PP NP formulations (Table 1). Transmission electron microscopy (TEM) further validated these findings, demonstrating spherical nanoparticles with uniform morphology (Figure 2C). Surface charge analysis via zeta potential measurements yielded values of −30.43 ± 1.76 mV (B-PP NPs) (Table 1) and −27.10 ± 1.18 mV (NM-PP NPs), suggesting colloidal stability (Figure 2D). Long-term stability assessments conducted over 18 days at both room temperature and 4 °C confirmed that particle size and PDI remained unchanged, indicating robust formulation integrity of NM-PP NPs (Figure 2E). Finally, in vitro drug release profiling demonstrated that NM-PP NPs exhibited sustained release kinetics, which was best described by the Korsmeyer–Peppas model (R^2^ = 0.99, *n* = 0.28, Table S1) [52]. Continuous release of NM was observed over 108 h, highlighting their potential for controlled drug delivery applications (Figure 2F).

### 3.2. Preparation and Characterization of ACE2 Decoy-Modified NM-PP NPs

#### 3.2.1. Conjugation of CTC-445.2d Protein to NM-PP NPs

**Figure 3:**
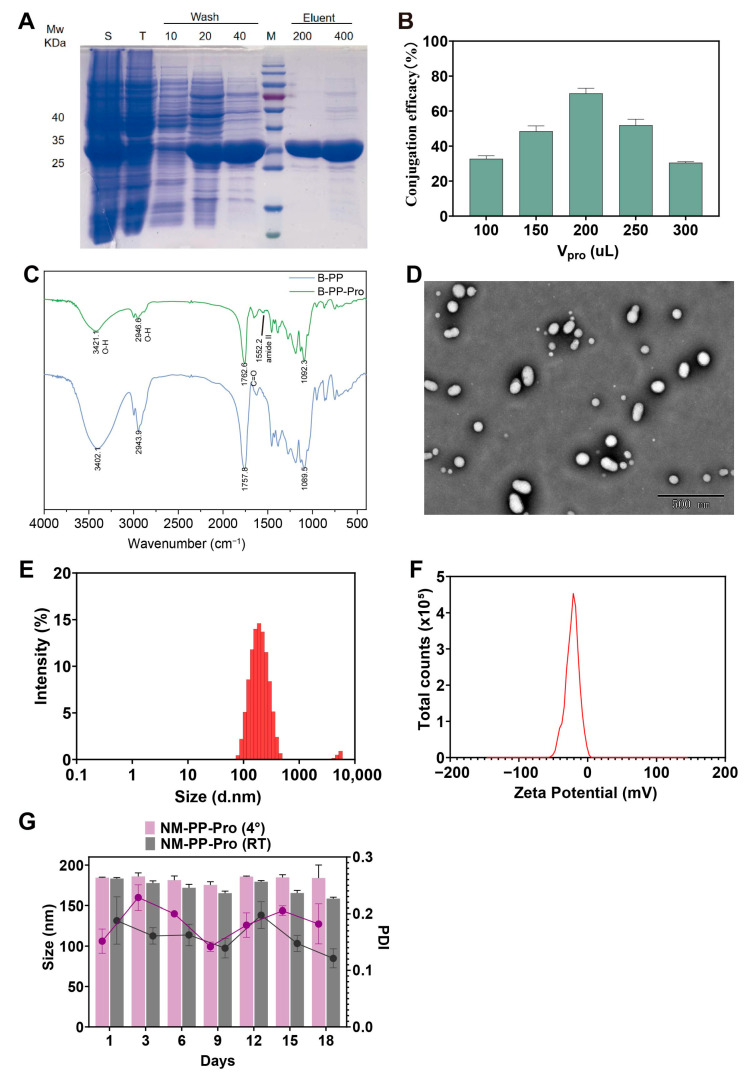
Preparation of CTC-445.2d protein and physical characterization of NM-PP-Pro NPs. (**A**) SDS-PAGE result of CTC-445.2d protein. (**B**) Conjugation efficiency of NM-PP-Pro NPs. (**C**) FT-IR spectra of B-PP NPs and B-PP-Pro NPs. (**D**–**F**) Hydrodynamic diameter (**D**) distribution, TEM (**E**), and zeta potential (**F**) of NM-PP-Pro NPs. (**G**) The particle size changes of NM-PP-Pro NPs investigated after placed at 4 °C and RT for 18 days (mean ± s.d., *n* = 3).

The recombinant ACE2 decoys (CTC-445.2d protein), featuring a C-terminus six-His-tag, was successfully expressed in *Escherichia coli* BL21(DE3) using the expression vector pET-29b (+). Protein expression was induced with 0.5 mM IPTG at 16 °C for 15 h, resulting in a high-level production of soluble recombinant protein. Following cell lysis using a high-pressure cell homogenizer, the soluble fraction was isolated by centrifugation. The target protein was subsequently employing a stepwise imidazole gradient (200–400 mM) for elution. SDS-PAGE analysis of the purified product revealed a single band at the predicted molecular weight of approximately 36 kDa (Figure 3A), confirming the successful isolation of the homogeneous CTC-445.2d protein. These findings are in agreement with a previous report on the de novo design and recombinant expression of CTC-445.2d [35], further validating our purification strategy.

The amino groups of ACE2 decoy protein CTC-445.2d were covalently conjugated to the terminal carboxylic acid groups of PP NPs via EDC/NHS-mediated amidation chemistry. Unmodified nanoparticles (B-PP NPs) served as controls to assess functionalization efficiency. Quantitative analysis revealed a coupling efficiency of 73.48% when using 200 μL of 1 mg/mL CTC-445.2d protein (Figure 3B). FT-IR spectroscopy analysis further confirmed successful conjugation (Figure 3C). Comparative evaluation of B-PP NPs and CTC-445.2d protein-conjugated nanoparticles (PP-Pro NPs) demonstrated the following distinct spectral shifts: B-PP NPs exhibited a broad O–H stretch at ~3400 cm^−1^ (carboxylic acid) and a strong C=O stretch at 1758 cm^−1^. Following conjugation, attenuation of the carboxylic acid signal and emergence of a new amide II band (1550 cm^−1^) confirmed covalent amide bond [48,53,54] formation between protein CTC-445.2d and PLGA-PEG-COOH, suggesting their successful conjugation.

NM-PP-Pro NPs retained a spherical morphology with a uniform size distribution (Figure 3D). Post-conjugation, the hydrodynamic diameter increased significantly from 157.3 ± 1.43 nm (NM-PP NPs) (Table 1 to 184.4 ± 0.62 nm (NM-PP-Pro NPs) (Figure 3E), consistent with the successful surface modification of NPs. The zeta potential also shifted from −27.10 ± 1.18 mV to −22.13 ± 0.06 mV (Figure 3F and Table 1), further supporting protein attachment. Over an 18-day stability study at both 4 °C and room temperature storage, no significant change in particle size was observed, and PDI always remained below 0.3, with only minor fluctuations (<0.28) at room temperature, suggesting the excellent physical stability of NM-PP-Pro NPs (Figure 3G). These results confirm that CTC-445.2d protein-conjugated nanoparticles NM-PP-Pro NPs maintain monodispersity, structural integrity, and long-term stability, making them suitable for further biological applications.

#### 3.2.2. Conjugation of Ultrashort Peptide (SI5α) to NM-PP NPs

**Figure 4:**
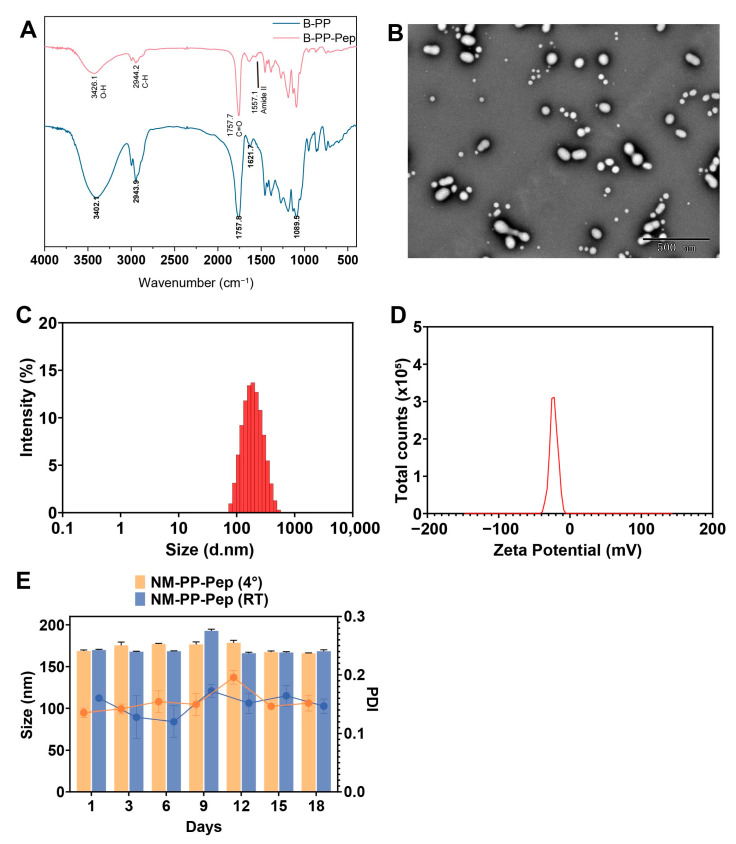
Physical characterization of NM-PP-Pep NPs. (**A**) FT-IR spectra of B-PP NPs and B-PP-Pep NPs. (**B**–**D**) TEM (**B**), hydrodynamic diameter distribution (**C**), and zeta potential (**D**) of NM-PP-Pep NPs. (**E**) The particle size changes of NM-PP-Pro NPs investigated after placed at 4 °C and RT for 18 days (mean ± s.d., *n* = 3).

We additionally functionalized the nanoparticles with SI5α, a computationally designed heptapeptide ACE2 decoy derived from the S protein binding domain of ACE2. FT-IR spectroscopy analysis revealed a new absorption peak at 1557 cm^−1^, corresponding to the characteristic amide II band (resulting from coupling between N-H bending and C-N stretching vibrations). This evidence confirmed the successful formation of an amide bond (-CONH-) between carboxylic acid and the amino group (Figure 4A), indicating the covalent conjugation of SI5α to PLGA-PEG-COOH (designated as PP-Pep NPs).

The SI5α modification of NM-PP NPs (NM-PP-Pep NPs) retained a spherical morphology with a uniform size distribution (Figure 4B). DLS measurements showed that NM-PP-Pep NPs induced a significant size increase to approximately 168.7 ± 1.25 nm (Figure 4C). This size was slightly larger than unmodified NM-PP NPs (157.3 ± 1.43 nm) but smaller than NM-PP-Pro NPs (184.4 ± 0.62 nm). The zeta potential showed a modest increase to about −27.47 ± 0.21 mV (Figure 4D) compared to NM-PP-Pro NPs (−22.13 ± 0.06 mV) and no increase compared to NM-PP NPs (−27.10 ± 1.18 mV).

In the 18-day stability assessments, NM-PP-Pep NPs maintained excellent physical stability at both 4 °C and room temperature storage conditions (Figure 4E). The hydrodynamic diameter remained constant throughout the observation period, with PDI consistently below the threshold value of 0.3. These findings confirm that NM-PP-Pep NPs have good monodispersity and storage stability.

### 3.3. Neutralization of SARS-CoV-2 Pseudovirus by Modified NM-PP NPs

Based on the physicochemical characterization of modified NM-PP NPs, this study further evaluated their antiviral efficacy in a susceptible 293T-ACE2 overexpression cell model. In the wild-type SARS-CoV-2 pseudovirus infection model, both free ACE2 decoys (CTC-445.2d protein and SI5α short peptide) and their corresponding functionalized NM-PP NPs (NM-PP-Pro NPs and NM-PP-Pep NPs) were pre-incubated with pseudovirus for 1 h before inoculation into the host cell culture system as reported [35,51]. Following 24 h of infection, fresh medium was added and incubation continued for 24 h. Viral infection levels were quantitatively assessed through chemiluminescence detection.

**Figure 5:**
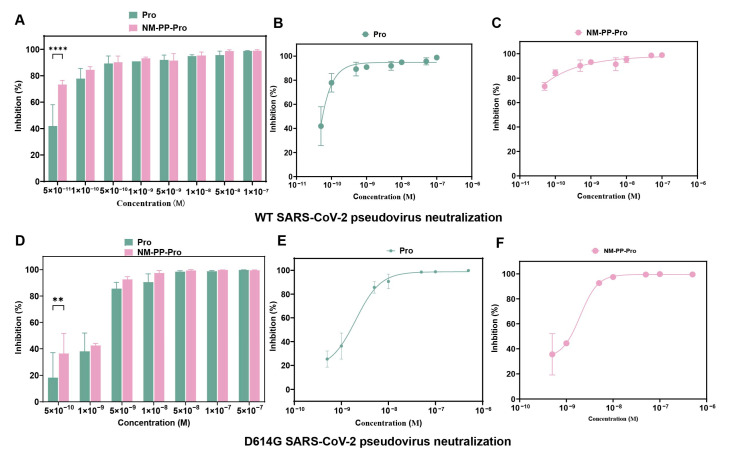
Inhibition of SARS-CoV-2 infection by CTC-445.2d protein and NM-PP-Pro NPs. (**A**) Neutralization of wild-type virus by nanoparticles at various concentrations. IC50 of the protein (**B**) and NM-PP-Pro NPs (**C**) against wild-type SARS-CoV-2. (**D**) Neutralization of D614G mutant virus by nanoparticles at various concentrations. IC50 of the protein (**E**) and NM-PP-Pro NPs (**F**) against D614G mutant ** *p* < 0.01, **** *p* < 0.0001).

CTC-445.2d has been previously shown to exhibit potent inhibitory activity against wild type SARS-CoV-2 in Calu-3 cells, with reported IC_50_ values below 5 nM [35]. Consistent with these findings, our current study confirms its efficacy in inhibiting wild-type pseudovirus infection in 293T cells at even lower concentrations (IC_50_ < 0.01 nM) (Table S2). NM-PP-Pro NPs also maintained antiviral activity comparable to free CTC-445.2d protein in the low nanomolar concentration range (0.1–100 nM), with no statistically significant difference observed (*p* > 0.1) (Figure 5A). Impressively, both NM-PP-Pro NPs (IC_50_ < 0.05 nM) and free CTC-445.2d protein exhibited exceptional viral inhibition, achieving over 85% suppression of host cell invasion at an ultralow concentration of 0.5 nM (Figure 5B,C).

Further evaluation using a D614G mutant pseudovirus model revealed distinct neutralization profiles. In contrast to the wild-type model where over 85% inhibition was achieved at 1 nM (Figure 5A), the mutant required higher concentrations for effective neuralization. While NM-PP-Pro NPs (IC_50_ = 1.973 nM) and free CTC-445.2d protein (IC_50_ = 2.023 nM) showed reduced efficacy (<50% inhibition) at 1 nM against the D614G mutant, dose optimization to 10 nM significantly enhanced the inhibition rate to >80% (Figure 5D–F and Table S2). These results indicate that, although the D614G mutant exhibits decreased sensitivity to NM-PP-Pro NPs compared to the wild-type virus, appropriate dosage adjustment can overcome this reduced susceptibility. This finding underscores the potential of NM-PP-Pro NPs in addressing viral mutation-induced drug resistance.

**Figure 6:**
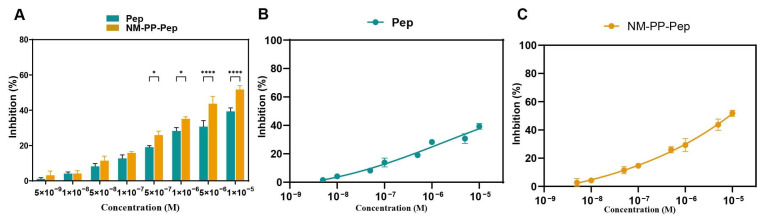
Inhibition of SARS-CoV-2 infection by SI5α peptide and NM-PP-Pep NPs. (**A**) Neutralization of wild-type virus by nanoparticles at various concentrations. IC50 of the peptide (**B**) and NM-PP-Pep NPs (**C**) against wild-type SARS-CoV-2 (* *p* < 0.05, **** *p* < 0.0001).

The ultrashort peptide SI5α has been shown to inhibit SARS-CoV-2 like coronavirus, with an EC_50_ value of 1.59 uM [33]. However, in our study, at a higher concentration of 100 μM, NM-PP-Pep NPs showed only moderate antiviral efficacy, with 51.77% inhibition of viral infection, representing a 1.32-fold improvement over free SI5α (IC_50_ > 10 μM) at the same concentration (39.32%) (Figure 6A–C). Given the limited viral neutralization capacity of NM-PP-Pep NPs, further investigation will prioritize the NM-PP-Pro NPs for further evaluation.

### 3.4. Nanoparticles-Cell Interaction Studies

**Figure 7:**
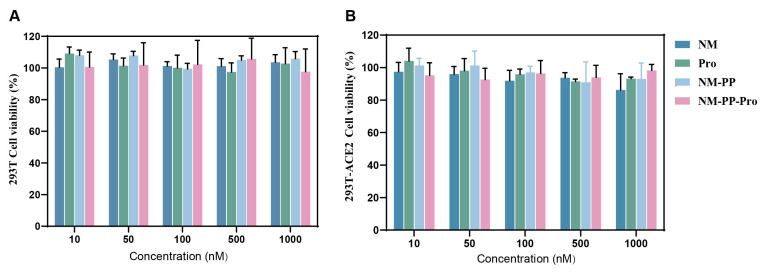
The concentration-dependent cytotoxicity of NM, Pro, NM-PP NPs, and NM-PP-Pro NPs in 293T cell (**A**) and 293T-ACE2 cell (**B**). The cells’ viability was determined by means of the CCK-8 assay and is expressed as the ratio of non-treated cells.

The cytotoxic effect of NM-PP-Pro NPs was evaluated in two mammalian cell lines, which were wild-type 293T and ACE2-overexpressing 293T cells, using the CCK-8 viability assay. In human embryonic kidney wild-type 293T cells, cell viability consistently retained above 80% at all tested concentration (10–1000 nM) of NM-PP-Pro NPs following 24 h of exposure (Figure 7A), suggesting the excellent biocompatibility of NM-PP-Pro NPs. Notably, this favorable safety profile was similarly demonstrated in ACE2-overexpressing 293T cells, where neither NM-PP NPs nor NM-PP-Pro NPs provoked any significant cytotoxic response. Cell viability metrics consistently exceeded 80% across all treatment groups at equivalent concentrations (Figure 7B), demonstrating the system’s robust biocompatibility in both cellular models.

**Figure 8:**
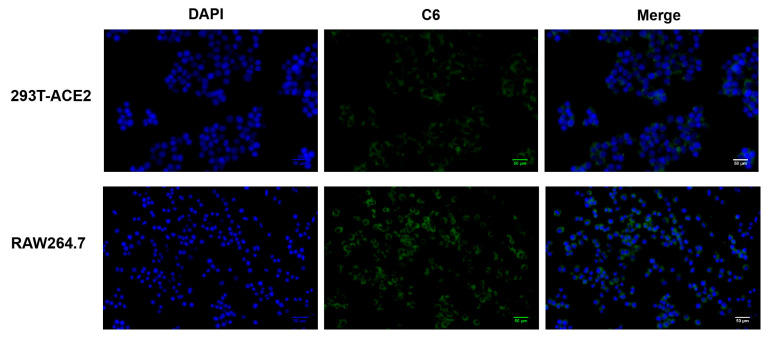
Visualization of 6-Coumarin-labeled (green) NM-PP-Pro NPs in 293T-ACE2 and RAW 264.7 cells (blue) via fluorescence microscope.

To investigate the intracellular distribution of NM-PP-Pro NPs, the nanoparticles were fluorescently labeled with Coumarin 6 for visualization. Following a 3 h incubation period, fluorescence microscopy analysis revealed robust cellular uptake in both 293T and RAW264.7 cell lines, with distinct perinuclear localization of green fluorescence (Figure 8). DAPI counterstaining confirmed that nanoparticle-derived fluorescence was predominantly localized in the cytoplasm, encircling the blue-stained nuclei (Figure 7). These findings demonstrate efficient cellular internalization and a clear cytoplasmic distribution pattern of NM-PP-Pro NPs.

## 4. Discussion

SARS-CoV-2 invades host cells by binding its S protein to the ACE2 receptor on the target cell surface. This interaction induces ACE2 downregulation upon infection of alveolar cells [55], disrupting the renin-angiotensin system (RAS) balance and overactivating the ACE-Ang II axis [56,57]. Elevated Ang II promotes macrophage infiltration and stimulates excessive production of pro-inflammatory cytokines (e.g., IL-6, MCP-1) and adhesion molecules (e.g., VCAM-1) [58], ultimately contributing to endothelial dysfunction. Concurrently, reduced ACE2 expression diminishes its protective role in lung tissue, increasing pulmonary capillary permeability and edema risk [59,60,61]. Thus, blocking S-ACE2 binding represents a promising strategy to mitigate ACE2-mediated inflammatory cascades and subsequent lung injury. Additionally, Niemeyer et al. demonstrated that nafamostat (NM) not only inhibits SARS-CoV-2 viral shedding in a human bronchiolar epithelial cell model, but it also suppresses baseline cytokine secretion even in the absence of viral infection, highlighting its intrinsic anti-inflammatory properties [62,63]. Based on the existing literature, we engineered ACE2 decoy-conjugated nanoparticles loaded with NM, which may offer dual therapeutic benefits, such as the competitive inhibition of viral entry and modulation of inflammatory responses to alleviate cytokine storm-associated damage. However, the anti-inflammatory effects require further experimental validation.

In this study, the following two nanoparticle formulations were developed: PLGA nanoparticles encapsulating nafamostat (NM-PP NPs) and NM-loaded PLGA nanoparticles conjugated with ACE2 decoys (NM-PP-Pro NPs and NM-PP-Pep NPs). Although NM has been previously reported as a potential inhibitor of TMPRSS2-a key protease mediating SARS-CoV-2 entry [45,49,64], its inclusion did not significantly enhance the antiviral efficacy of ACE2 decoy-conjugated nanoparticles (NM-PP-Pro NPs and NM-PP-Pep NPs) compared to ACE2 decoy-conjugated NPs without loading NM (Figure S1, Table S2). Given that NM release from NPs was confirmed to be efficient (Figure 2F), the lack of NM-mediated antiviral enhancement in ACE2-overexpressing 293T cells may be due to the negligible endogenous TMPRSS2 expression in this cellular model (Figure S2; Human Protein Atlas). The discrepancy underscores the importance of utilizing cell models co-expressing both ACE2 and TMPRSS2 (Vero E6 or Calu-3) to properly evaluate the potential synergistic effects between NM and ACE2 decoys in the NM-PP-Pro NP system, which will be the focus of our future investigations.

A central innovation of our platform lies in its dual-functional design based on PLGA-PEG carriers, which simultaneously encapsulate NM and present high-affinity ACE2-mimetic decoys on their surface. Unlike other reported ACE2-modified nanoparticles, our system integrates both inhibitory and neutralization functions in a single entity. The decoys employed here represent significant advances; CTC-445.2d is a rationally engineered ACE2 variant with reduced molecular weight, improved stability, and enhanced neutralizing potency across multiple cellular models [35]. Similarly, SI5α is a computationally designed heptapeptide that can be efficiently synthesized chemically and demonstrates moderate antiviral activity in SARS-CoV-2 infection models [33]. As expected, CTC-445.2d-modified NM-PP-Pro NPs demonstrated potent neutralizing activity against pseudo-typed SARS-CoV-2 in vitro (IC_50_ < 0.05 nM), while NM-PP-Pep NPs modified by SI5α showed limited antiviral activity (IC_50_ > 10 μM) (Table S2).

In addition to antiviral performance, we also assessed preliminary biocompatibility. ACE2-decoy-modified nanoparticles (NM-PP-Pro/Pep NPs) showed excellent cytocompatibility at a therapeutically relevant concentration (1 μM), maintaining cell viability above 85% in CCK-8 assays. The use of FDA-approved PLGA-PEG as the carrier material further supports the favorable safety profile of the system due to its well-documented biocompatibility and low toxicity [65]. NM itself also has an established clinical safety record. However, critical parameters such as hemocompatibility remain to be assessed and will be systematically investigated in follow-up studies.

In summary, we successfully developed ACE2-decoy-modified nanoparticles (NM-PP-Pro/Pep NPs) by encapsulating the therapeutic agent NM and conjugating the surface with the specific ACE2 decoys (CTC-445.2d or SI5α). Both unmodified NM-PP NPs and ACE2-decoy-modified NM-PP-Pro/Pep NPs exhibited a uniform size distribution with the average diameters below 200 nm and a negatively charged surface, as confirmed by zeta potential analysis. Stability assessments confirmed that these NPs retained structural integrity for at least 18 days under storage conditions at 4 °C and room temperature. In vitro release profiles further indicated sustained and controlled nafamostat release. Notably, NM-PP-Pro NPs exhibited potent antiviral efficacy, with an IC_50_ value below 0.05 nM against wild type SARS-CoV-2. Moreover, NM-PP-Pro NPs maintained nanomolar scale efficacy against the D614G mutant of SARS-CoV-2 (IC_50_ = 2 nM). These findings highlight the potential of NM-PP-Pro NPs as a versatile nanotherapeutic platform for targeting SARS-CoV-2 and its emerging variants.

## Figures and Tables

**Table 1:** Physicochemical properties of B-PP, NM -PP, NM-PP-Pro, and NM-PP-Pep.

Name	Size (nm)	Zeta (mV)	PDI	EE (%)	LD (%)
B-PP	155.4 ± 0.81	−30.433 ± 1.76	0.156 ± 0.02	\	\
NM-PP	157.3 ± 1.43	−27.100 ± 1.18	0.152 ± 0.213	74.93 ± 2.18%	3.44 ± 0.17%
NM-PP-Pro	184.4 ± 0.62	−22.133 ± 0.06	0.152 ± 0.021	70.51 ± 1.78%	3.74 ± 0.42%
NM-PP-Pep	168.7 ± 1.25	−27.467 ± 0.21	0.135 ± 0.078	69.21 ± 2.45%	3.34 ± 0.29%

## Data Availability

The original contributions presented in this study are included in the article. Further inquiries can be directed to the corresponding author.

## Supplementary Materials

The following supporting information can be downloaded at: https://www.mdpi.com/article/10.3390/v17091167/s1, Figure S1: In vitro wild-type SARS-CoV-2 pseudovirus neutralization; Figure S2: The expression of TMPRSS2 in different cells; Table S1: Kinetic release parameters for NM-PP NPs. Table S2: The half maximal inhibitory concentration (IC_50_) of nanoparticles.

## Abbreviations

The following abbreviations are used in this manuscript:

ACE2	angiotensin-converting enzyme 2
NM	nafamostat
TMPRSS2	transmembrane serine protease 2
NP	nanoparticle
PLGA-PEG	Poly(lactide-co-glycolide)-poly (ethylene glycol)
IPTG	isopropyl-β-D-thiogalactopyranoside
EDC	1-(3-Dimethylaminopropyl)-3-ethylcarbodiimide hydrochloride
NHS	N-Hydroxysuccinimide
PDI	polydispersity index
