# Supplementary material for: ACE2-Decoy-Conjugated PLGA-PEG Nanoparticles Loaded with Nafamostat for Potent Antiviral Activity
Source: Viruses. 2025 Aug 27;17(9):1167. doi: 10.3390/v17091167 (PMC12474491; doi:10.3390/v17091167)
Supplement: Supplementary file 1 [file viruses-17-01167-s001.zip › viruses-3793704-supplementary.pdf]

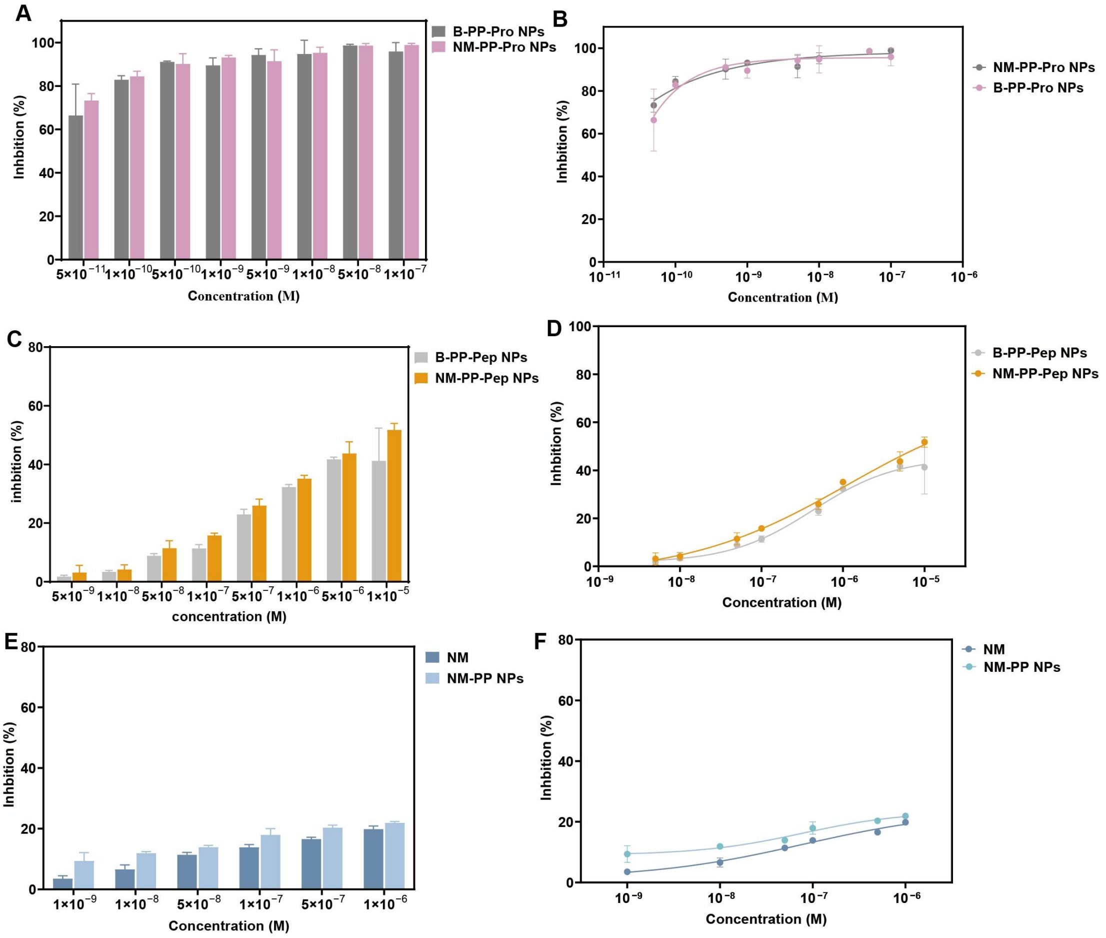


**Supplementary Figure S1.** In vitro wild-type SARS-CoV-2 pseudovirus neutralization

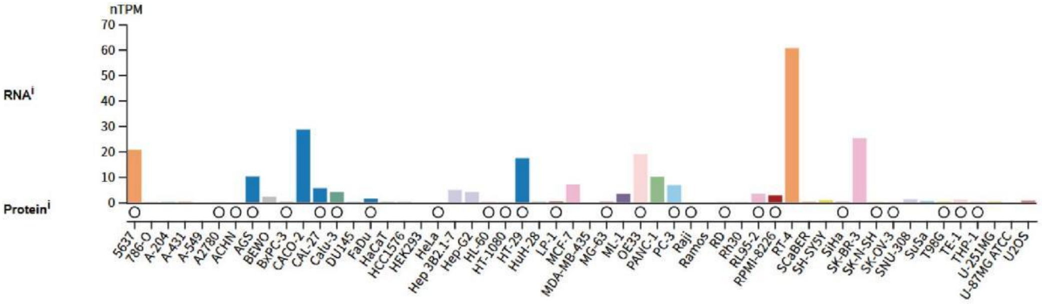


**Supplementary Figure S2.** The expression of TMPRSS2 in different cells

**Table S1.** Kinetic release parameters for NM-PP NPs.

| Mathematical model | Parameter | Value | $R^2$ |
|---|---|---|---|
| Korsmeyer-Peppas | K | 9.59 | 0.99 |
| | n | 0.28 | |
| Higuchi | $K_h$ | 2.47 | 0.98 |
| Zero-Order | $K_0$ | 0.20 | 0.91 |
| First-Order | $K_1$ | 29.90 | 0.80 |

**Table S2.** The half maximal inhibitory concentration ($IC_{50}$) of nanoparticles.

| SARS-CoV-2 pseudovirus | $IC_{50}$ (nM) | |
|---|---|---|
| | WT | D614G |
| NM | ＞1000 | ＞1000 |
| CTC-445.2d protein | ＜0.01 | 2.02 |
| SI5α peptide | ＞1000 | - |
| NM-PP NPs | ＞1000 | - |
| B-PP-Pro NPs | ＜0.05 | 2.55 |
| NM-PP-Pro NPs | ＜0.05 | 1.97 |
| B-PP-Pep NPs | ＞1000 | - |
| NM-PP-Pep NPs | ＞1000 | - |
